# Effects of weekly pain monitoring on back pain outcomes: a non-randomised controlled study

**DOI:** 10.1186/s12998-021-00393-2

**Published:** 2021-09-16

**Authors:** Alice Kongsted, Tue Secher Jensen, Klaus Doktor, Lise Hestbæk

**Affiliations:** 1 Chiropractic Knowledge Hub, Campusvej 55, 5230 Odense M, Denmark; 2 Department of Sports Science and Clinical Biomechanics, University of Southern Denmark, Campusvej 55, 5230 Odense M, Denmark; 3 Diagnostic Center, Silkeborg Regional Hospital, Falkevej 1, 8600 Silkeborg, Denmark

**Keywords:** Back pain, Data collection, Monitoring, Pain measurement, Self-management

## Abstract

**Background:**

Disease monitoring is an important element of self-management of several chronic diseases. Pain monitoring has become very easily available, but the role in musculoskeletal pain conditions is not clear. Awareness of pain might be helpful for people to understand pain, but focusing on pain may on the contrary negatively affect pain experience and behaviours. The objective of this study was to investigate the potential impact of pain monitoring on low back pain (LBP), specifically to determine if pain intensity, activity limitation and pain control, differed between patients with weekly pain monitoring over 12 months and patients with follow-ups at 2 weeks, 3 months and 12 months.

**Methods:**

This was a non-randomised controlled study embedded in a cohort study with data collection November 1st 2016 to December 21st 2018. Adults seeking care for LBP were enrolled at the first visit to a chiropractor and followed with surveys after 2 weeks, 3 months and 12 months. Those enrolled first, n = 1,623, furthermore received weekly SMS-questions about pain frequency and pain intensity, whereas those enrolled next was the control group, n = 1,269 followed only by surveys. Outcomes at 12-months were compared, adjusting for group differences on baseline parameters.

**Results:**

LBP intensity (0–10) was slightly lower at 12-months follow-up in the SMS group than the control group (adjusted beta − 0.40 (95% CI: − 0.62; − 0.19)). No relevant between-group differences were observed for activity limitation (0–100) (1.51 (95% CI: − 0.83; 3.85)) or ability to control pain (0–10) (− 0.08 (95% CI − 0.31; 0.15)).

**Conclusions:**

Frequent pain monitoring did not demonstrate any negative effects of weekly pain monitoring, and it was perhaps even helpful. The role of self-monitoring as part of self-managing LBP should be explored further including optimal frequencies, formats, and methods for feedback.

***Trial registration*:**

The study was not registered as a clinical trial.

## Background

Non-communicable diseases constitute a large part of the global burden of disease with musculoskeletal conditions being among the main causes for disability [1, 2]. People living with persistent or recurrent conditions need knowledge, skills, and tools to manage their health conditions in everyday life [3]. For many people this involves contact with health care providers, but most of the time people manage health conditions on their own or with assistance from family and friends.

Self-management may involve engagement in health promoting activities, symptom management and self-monitoring of the condition [4]. Self-monitoring of physiological measures such as blood glucose levels in diabetes and blood pressure in hypertension are important for individual health decisions. In musculoskeletal pain conditions, the role of disease monitoring is less clear, as there are no objective measures to inform actions. Musculoskeletal pain often varies considerably from day to day, and monitoring may be helpful for people to make sense of their pain [5]. Also, frequent pain measures are used in research to evaluate the course of pain as trajectories of pain are not well captured by the few follow-ups commonly used [6, 7].

Self-monitoring of pain has become easily available and was an integrated feature in 11 out of 19 and 14 out of 28 pain management apps identified in two recent reviews [8, 9]. This awareness of pain might be helpful for people to understand the variation over time and reflect on how the pain relates to other aspects of life [5]. However, a continuous focus on pain might also negatively affect pain intensity and pain behaviours, as expecting pain and focusing on pain entail a potential for affecting pain perception negatively [10]. It is thus important to consider if self-monitoring as part of interventions could be harmful. In research it is also important to know if the frequent measuring of pain affects outcomes differently than using few follow-ups over the same time period.

Currently there is very sparse evidence of the potential effects of pain monitoring on patient outcomes. A systematic review of effectiveness of digital support interventions for self-management of back pain did not identify studies providing evidence for or against self-monitoring of pain [11]. In a previous explorative study, we reported outcomes in patients with back pain and in school children who participated in weekly SMS-monitoring of pain as compared to similar samples with single time point follow-ups, and observed slightly better outcome with weekly pain measurements than in those without. However, there was a considerable risk that samples were not comparable on unmeasured factors [12].

The objective of this study was to determine if outcomes, in terms of pain intensity, LBP related activity limitation and pain control, differed between patients with weekly pain monitoring over 12 months and patients with follow-ups at 2 weeks, 3 months and 12 months only.

## Method

This was a non-randomised controlled study embedded in the Danish Chiropractic Low Back Pain Cohort (ChiCo). All patients included were invited to complete baseline and follow-up questionnaires (see "Data Collection" below). Weekly SMS-tracking of pain in addition to follow-up questionnaires was planned for the first patients enrolled in the cohort until a level of 1,000 participants actively answering the text messages was reached, while the subsequent participants were followed by questionnaires only. Treatment was not affected by study participation, and the treating chiropractors were blinded to the patient reported information registered in the study. The ChiCo cohort and procedures for recruitment and data collection have previously been described in detail [13]. The protocol for this study was not pre-registered. All methods were carried out in accordance with relevant guidelines and regulations.

### Setting

Participants were recruited from a convenience sample of ten Danish chiropractic clinics. Most patients self-refer to chiropractic treatment in Denmark. Around 20% of costs of chiropractic care is covered by the national health insurance, while the rest is paid out of pocket, by the patients’ private insurance, or some combination of both.

### Participants

Adults (> 18 years of age) consulting with a primary complaint of LBP with or without leg pain were eligible for inclusion. People who either could not read Danish, were in ongoing treatment for LBP, or with suspected serious pathology were not invited to the study.

### Allocation to SMS-tracking

**Fig. 1:**
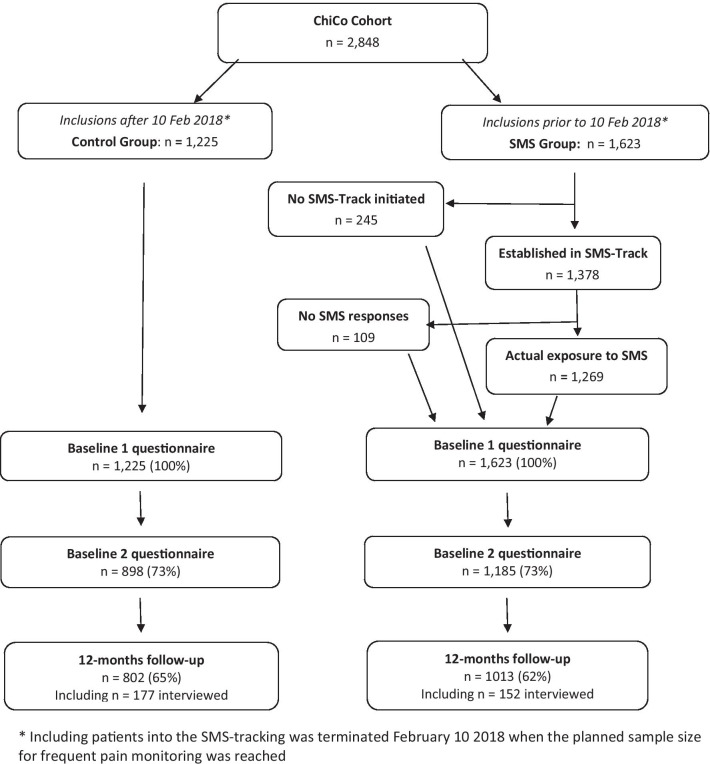
Study flowchart

Based on a sample size estimation for investigating trajectories of LBP (unrelated to this study), 1,000 participants with weekly data collection using SMS-tracking was needed. Because of considerable costs related to the set-up of SMS-tracking, this was planned for the participants first enrolled, and to compensate for project participants without a mobile phone and expected drop-outs, 1,623 were enrolled in the SMS-tracking sample (hereafter referred to as the SMS group). Those recruited subsequently were not followed with SMS-tracking and constituted the control group (Fig. 1). We included the entire sample in the study which would be sufficient to demonstrate even a very small effect size.

### Content of SMS-tracking

The SMS group received text messages to their mobile phone every week starting seven days after enrolment. The questions asked were (1) “How many days have you had back pain (or back-related leg pain) within the last 7 days? (please answer with one number from 0 to 7)”; (2) “How severe was the pain typically on a scale from 0 to 10?”; and (3) “How many days were you home last week from work or study because of your back pain? (please answer with one number from 0 to 7)”. In case the answer to the first message was “0” the two following messages were not sent. If no response was registered within 2 days, participants received one automated reminder.

### Data collection in both groups

Questionnaire data were collected and stored using the online system Research Electronic Data Capture (REDCap) hosted by the Odense Patient data Explorative Network (OPEN). Record IDs and mobile phone numbers were exported to an SMS-Track service that automated weekly distribution of text message questions. Baseline data was collected in two parts. The initial part via an iPad in the clinic prior to the consultation and the second part via a link to an online survey sent by e-mail to the participant on the day of enrolment. Links to follow-up questionnaires were sent after 2 weeks, 3 months, and 12 months. Timing and content of the questionnaires did not differ between the SMS group and the control group.

### Outcome measures

The primary outcome measure for this study was LBP intensity at 1-year follow-up. Activity limitation and pain control after 1 year were secondary outcomes.

LBP intensity was measured on a 0–10 numeric rating pain scale (0 = no pain; 10 = worst imaginable pain) asking about typical pain in the previous week [14, 15]. Moderate to severe pain at follow-up was defined as NRS > 3 [16].

Activity limitation was measured by the 23-item Roland Morris Disability Questionnaire (RMDQ) converted to a proportional sum-score ranging from 0 to 100 (0 = No disability; 100 = Fully disabled) [15, 17]. Missing items in partly completed RMDQs were imputed by multiple imputation prior to calculating the sum-score. Non-improvement on RMDQ was defined as < 30% reduction in the sum score from baseline to 1-year follow-up [18].

Pain control was measured by a single coping item from the Örebro Musculoskeletal Pain Questionnaire (ÖMPQ) (Danish version) asking “Given an average day, to what extent can you handle or control your pain?” (0 = Not at all; 10 = Complete control) [19]. As no formal cut-point exists for categorisation of the item, we defined “lack of control” as scoring < 5. The ÖMPQ was developed as a prognostic screening tool and this measure of pain control has not been validated as an outcome measure.

### Baseline variables

The two study groups were compared at baseline on the following parameters in addition to baseline values of outcome measures: Age (years), sex, education (higher or further education, vocational education, no qualifying education, other education), physical load at work (very strenuous, strenuous, somewhat strenuous, light, very light), episode duration, STarT Back Tool risk profile (low, medium, high risk), and recovery expectation (ÖMPQ “How certain are you that you will be able to return to ALL of your usual activities 1 month from today?”).

### Analyses

Patient characteristics were described as means with standard deviations (SD) and proportions (%).

Potential differences between the SMS group and the control group in the characteristics of those not completing 12-months follow-up were investigated in logistic regression models with SMS group, baseline characteristic (age, sex, LBP intensity, activity limitation, pain control, STarT risk profile, episode duration, recovery expectations) and an interaction between the two as independent variables and drop out as the dependent variable.

Group differences were investigated using the “intention to treat” principle, with all patients invited to SMS-tracking analysed in the SMS group even if not responding to any SMS questions. In a sensitivity analysis we included only those responding to SMS in more than 26 weeks, i.e. more than half of the SMS follow-ups, in the SMS group. The control group did not differ between the two analysis.

Group differences were estimated by linear (continuous outcomes) and logistic (binary outcomes) mixed models with clinician as random effects to account for dependence between observation from the same chiropractor and reported as beta coefficients and Odds Ratios (OR) with 95% confidence intervals (CI). Group differences were reported as crude estimates and after adjustment for baseline values that were observed to differ slightly between groups: LBP intensity, age, sex, duration of present episode, educational level, and workload.

Analyses were performed using Stata 16.1 (StataCorp, Texas, USA).

## Results

### Study participants and response rates

ChiCo cohort recruitment was initiated on November 1st, 2016. Participants enrolled prior to February 10th, 2018 totaling 1,623, formed the SMS group, while the remaining 1,225 participants enrolled until the cohort closure on December 21st, 2018 formed the control group.

Demographic parameters including age, sex, BMI and employment did not differ substantially between the groups (Table 1). Compared to the control group, a slightly larger proportion of participants in the SMS group reported a higher education (54% vs. 49%) a light or very light workload (62% vs. 58%), and more participants in the SMS group, and a very short duration of the present episode of pain (49 vs. 44%). The two groups had very similar baseline values of LBP intensity, activity limitation, pain control and recovery expectations (Table 1).

**Table 1:** Patient characteristics at baseline

Patient characteristics at baseline	SMS group (N = 1,623)	Control group (N = 1,225)
Age; mean (SD, range)	44.5 (13.6; 18–80)	44.7 (13.8; 18–87)
Sex, female; n (%)	655 (40.4%)	512 (41.8%)
BMI; mean (SD)	26.7 (4.9)	27.0 (5.1)
Employment, yes; n (%)	1302 (82.4%)	985 (81.8%)
*Longest education*		
Higher or further education; n (%)	615 (53.5%)	425 (48.8%)
Vocational education; n (%)	318 (27.7%)	276 (31.7%)
No qualifying education; n (%)	174 (15.1%)	139 (16.0%)
Other education; n (%)	43 (3.7%)	31 (3.6%)
*Workload*		
Very strenuous; n (%)	19 (2.1%)	18 (2.6%)
Strenuous; n (%)	108 (11.9%)	84 (12.1%)
Somewhat strenuous; n (%)	216 (23.8%)	186 (26.9%)
Light; n (%)	281 (30.9%)	225 (32.5%)
Very light; n (%)	285 (31.4%)	179 (25.9%)
Previous episodes with low back pain, yes; n (%)	934 (82.4%)	718 (84.4%)
Previous treatment for low back pain, yes; n (%)	763 (65.7%)	594 (67.4%)
Back pain intensity, NRS (0–10); mean (SD)	6.7 (2.1)	6.7 (2.1)
*Episode duration*		
1–7 days; n (%)	793 (49.4%)	535 (43.8%)
1–4 weeks; n (%)	359 (22.3%)	315 (25.8%)
1–3 months; n (%)	169 (10.5%)	163 (13.4%)
> 3 months; n (%)	286 (17.8%)	208 (17.0%)
*Prognostic index, STarT*^*a*^		
Low; n (%)	657 (42.3%)	513 (43.4%)
Medium; n (%)	558 (35.9%)	409 (34.6%)
High; n (%)	340 (21.9%)	260 (22.0%)
Activity limitation, RMDQ (0–100); mean (SD)^b^	55.6 (23.8)	54.1 (24.0)
Ability to control pain, NRS (0–10); mean (SD)	5.5 (2.4)	5.5 (2.3)
Recovery expectations, NRS (0–10); mean (SD)	7.4 (3.0)	7.5 (2.9)

*NRS*, Numeric Rating Scale;* RMDQ*, Roland Morris Disability Questionnaire;* STarT*,The Keele STarT Back Screening Tool;* SD*, Standard deviation

^a^Missings imputed if at least 7 out of 9 STarT items were answered.

^b^Missings imputed for partly completed RMDQ

In both groups, a response rate of 73% for the second part of the baseline questionnaire was obtained, while the SMS group had slightly lower response rates at 12-months follow (Fig. 1). Most baseline characteristics were not associated with non-response to 12-months follow-up, and the associations did not differ substantially between the groups (Table 2).

**Table 2:** Drop-out analysis

Patient characteristics at baseline	SMS group OR (95% CI) N = 1,013 responders N = 610 non-responders	Control group OR (95% CI) N = 802 responders N = 423 non-responders	Interaction between group and characteristic OR (p-value)
Age (years)	0.98 (0.97–0.99)	0.97 (0.96–0.98)	1.01 (p = 0.1)
Female (ref. male)	0.99 (0.80–1.21)	0.80 (0.63–1.02)	1.23 (p = 0.2)
Back pain intensity, NRS (0–10)	1.01 (0.96–1.06)	1.02 (0.96–1.08)	0.99 (p = 0.8)
Activity limitation, RMDQ (0–100)	1.00 (1.00–1.00)	1.00 (1.00–1.01)	1.00 (p = 0.8)
Ability to control pain, NRS (0–10)	0.92 (0.88–0.96)	0.94 (0.89–0.99)	0.98 (p = 0.5)
Recovery expectations, NRS (0–10)	0.98 (0.94–1.01)	0.98 (0.94–1.02)	0.99 (p = 0.8)
*STarT risk profile*			
Low (ref)			
Medium	1.09 (0.86–1.39)	0.95 (0.72–1.25)	1.15 (p = 0.4)
High	1.49 (1.14–1.95)	1.36 (1.00–1.85)	1.10 (p = 0.6)
*Episode duration*			
< 1 week (ref)			
1–4 weeks	0.92 (0.71–1.19)	0.96 (0.71–1.29)	0.95 (p = 0.8)
1–3 months	0.79 (0.55–1.12)	0.73 (0.50–1.08)	1.08 (p = 0.8)
> 3 months	1.13 (0.86–1.49)	1.41 (1.02–1.96)	0.80 (p = 0.3)

Odds ratios for non-response at 12 months follow-up for the SMS group and the control group

NRS = Numeric Rating Scale; RMDQ = Roland Morris Disability Questionnaire; STarT = The Keele STarT Back Screening Tool; OR = Odds ratio; CI = confidence interval; ref = reference group

### Effect of SMS-tracking

The primary outcome LBP intensity was slightly lower in the SMS group than the control group (mean 2.1 vs. 2.6; adjusted beta − 0.40 (95% CI: − 0.62; − 0.19)). This was also reflected in a lower proportion with pain intensity > 3 in the SMS group than in the control group (23.3% vs. 29.9%; adjusted OR 0.70 (95% CI: 0.56; 0.88)) (Tables 3 and 4). Secondary outcomes did not differ importantly between groups but showed some imprecision (Tables 3 and 4).

**Table 3:** Observed outcomes in the control group, the total SMS group, and the participants in the SMS group responding in more than 26 out of the 52 weeks

Continuous outcomes	Control group (N = 1,225)	SMS group (N = 1,623)	SMS group responding in > 26 weeks (N = 995)
LBP at 12 months follow-up, NRS (0–10); mean (SD)	2.6 (2.4) (n = 799)	2.1 (2.4) (n = 1,012)	2.0 (2.4) (n = 826)
Activity limitation, RMDQ (0–100); mean (SD)	19.5 (22.8) (n = 610)	20.1 (22.9) (n = 847)	19.9 (22.6) (n = 720)
Ability to control pain, NRS (0–10); mean (SD)	7.9 (2.4) (n = 790)	7.9 (2.6) (n = 999)	7.9 (2.6) (n = 813)

Binary outcomes	Control group (N = 1,225)	SMS group (N = 1,623)	SMS group responding in > 26 weeks (N = 995)
Pain > 3, yes; n (%)	239 (29.9%) (n = 799)	236 (23.3%) (n = 1,012)	182 (22.0%) (n = 826)
Non-improvement of activity limitation; n (%)	137 (22.8%) (n = 601)	184 (22.0%) (n = 835)	152 (21.3%) (n = 714)
Lack of pain control, yes; n (%)	85 (10.8%) (n = 790)	105 (10.5%) (n = 999)	90 (11.1%) (n = 81)

LBP = low back pain; NRS = Numeric Rating Scale; RMDQ = Roland Morris Disability Questionnaire; SD = standard deviation

**Table 4:** Group differences at 12-months follow-up

Continuous outcomes	SMS group versus control group Main analysis		SMS group responding > 26 weeks versus control group Sensitivity analysis	
	Crude β (95% CI)	Adjusted β (95% CI)	Crude β (95% CI)	Adjusted β (95% CI)
LBP at 12 months follow-up, NRS (0–10); mean (SD)	− 0.42 (− 0.65; − 0.19)	− 0.40 (− 0.62; − 0.19)	− 0.48 (− 0.73; − 0.24)	− 0.44 (− 0.68; − 0.21)
Activity limitation, RMDQ (0–100)	1.12 (− 1.34; 3.58)	1.51 (− 0.83; 3.85)	0.88 (− 1.68; 3.45)	1.30 (− 1.12; 3.73)
Ability to control pain, NRS (0–10)	− 0.05 (− 0.28; 0.19)	− 0.08 (− 0.31; 0.15)	− 0.10 (− 0.36; 0.15)	− 0.12 (− 0.37; 0.13)

Binary outcomes	Crude OR (95% CI)	Adjusted OR (95% CI)	Crude OR (95% CI)	Adjusted OR (95% CI)
Pain > 3	0.71 (0.58; 0.88)	0.70 (0.56; 0.88)	0.66 (0.53; 0.83)	0.65 (0.50; 0.83)
Non-improvement of activity limitation	1.00 (0.77; 1.31)	1.02 (0.77; 1.34)	0.94 (0.71; 1.24)	0.99 (0.72; 1.28)
Lack of pain control	0.97 (0.72; 1.32)	1.00 (0.74; 1.36)	1.03 (0.76; 1.41)	1.05 (0.76; 1.45)

Adjusted for back pain at baseline, age, sex, episode duration, education and workload. LBP = low back pain; NRS = Numeric Rating Scale; RMDQ = Roland Morris Disability Questionnaire; SD = standard deviation

### Sensitivity analysis

Of the 1,623 participants in the SMS group, 245 did not provide mobile phone information and another 109 did not answer any of the text messages. The group of participants actually responding to SMS tracking thus consisted of 1,269 participants of whom 995 (78%) answered more than 26 of the 52 text messages (median 51 responses (IQR 38–52)). Within the SMS group, we compared baseline characteristics of participants answering more than half the text messages, less than half, or none at all. Females, older participants, and patients with a short duration of the present episode, or a STarT low risk profile more often responded to more than half of the SMS questions, whereas baseline values of LBP intensity, activity limitation and ability to control pain were almost the same across the three responder groups (Table 5).

**Table 5:** Patient characteristics of the SMS group divided into subgroups responding to SMS questions in > 26 weeks (more than half of SMS messages); 1–26 weeks, 0 weeks

Patient characteristics at baseline	SMS group		
	> 26 responses (n = 995)	1–26 responses (n = 274)	0 responses (n = 354)
Age; mean (SD)	45.6 (13.0)	41.9 (14.8)	43.5 (14.2)
Sex, female; n (%)	420 (42.2%)	103 (37.6%)	132 (37.3%)
Back pain intensity, NRS (0–10); mean (SD)	6.7 (2.0)	6.7 (2.1)	6.6 (2.1)
Episode duration, > 7 day; n (%)	478 (48.6%)	148 (54.4%)	188 (53.6%)
*Prognostic index, STarT*			
Low; n (%)	428 (44.3%)	89 (33.8%)	140 (43.1%)
Medium; n (%)	351 (36.3%)	92 (35.0%)	115 (35.4%)
High; n (%)	188 (19.4%)	82 (31.2%)	70 (21.5%)
Activity limitation, RMDQ (0–100); mean (SD)	55.3 (23.7)	58.0 (23.3)	54.6 (24.3)
Ability to control pain, NRS (0–10); mean (SD)	5.5 (2.3)	5.3 (2.3)	5.4 (2.4)

NRS = Numeric Rating Scale; RMDQ = Roland Morris Disability Questionnaire; STarT = The Keele STarT Back Screening Tool; SD = standard deviation

Only including those who responded to more than 26 out of 52 SMS messages in the group comparisons revealed results similar to the intention to treat analysis (Tables 3 and 4).

## Discussion

### Principal findings

In this large non-randomised controlled study nested within the Danish ChiCo, we found a slightly lower pain intensity at 12 months follow-up in LBP patients who had provided weekly reports of their LBP through SMS-tracking, compared to patients who had not reported pain through SMS-tracking. The magnitude of the observed positive effect does not seem clinically relevant as a treatment effect, but provides evidence of weekly pain monitoring not having harmful effects on pain outcomes. There were no between-group differences observed in relation to activity limitation or pain control.

These findings align well with the results from a previous study also demonstrating lower odds for pain intensity above 3/10 at follow-up in the SMS-group than in the control group, but no differences in frequency, activity limitation and bothersomeness [12]. However, data used in that study were not optimally suited for the purpose of studying effects of frequent monitoring and the sample was small and suffered substantially from drop-out.

### Strengths and limitations

To our knowledge, this is the first cohort of patients with musculoskeletal pain which has sufficient power and a study design making it possible to compare two otherwise similar groups with and without frequent pain monitoring. There was no difference in attrition among the two groups, but the SMS group reported slightly higher education, lower workload, and shorter baseline duration of LBP than the control group. As all three of these have been shown to predict a better prognosis [20, 21], it cannot be ruled out that group differences reflected in these characteristics could explain (part of) the better pain outcome observed in the SMS group. However, we believe the influence of these factors are limited as the outcome (pain intensity, activity limitation and ability to control the pain) were similarly distributed among the two groups at baseline, and the analyses were adjusted for relevant baseline factors. We did not find matching of cases and controls justified as the potential advantages are likely to be minimal, since group allocation occurred through a non-selective chronological mechanism, and baseline differences between the two groups were minor.

The two groups were sampled in the same manner, since they were included in the same cohort, but the control group followed the SMS group in time. This time difference could potentially lower the credibility of comparisons but there is no reason to believe that this is the case, since there was no time gap between sampling of the two groups. Sampling simply continued, just without SMS-tracking, when the required number was reached for the SMS-tracking. Furthermore, there were no changes in treatment recommendations, reimbursement or administrative procedures in health care during the total sampling period (2016–2018).

### Implications for management

Remote patient monitoring is proposed as a tool to facilitate communication between patient and health care provider in order to improve timeliness, patient autonomy, and quality of care [22–24], and pain monitoring is common in pain management apps [8, 9]. However, in the case of LBP there is still not much evidence to guide a response to changes in pain reporting, although the field of remote monitoring and telemedicine is also expanding for LBP management [25, 26] and intelligent decision support systems are being developed [27]. For the time being, it seems that the simple advice to systematically monitor pain could potentially prove helpful for patients with LBP. It is possible that self-monitoring leads to self-reflection, which in turn might support helpful habits and facilitate positive behavior changes.[5]. However, despite emerging evidence for effectiveness of e-health supported self-management in LBP [28], currently there is no evidence to suggest how the element of pain tracking may improve self-management and health care for LBP. Our results support continued research into this field as the intervention seems free of general negative side effects.

### Implications for research

Continuous registration of signs and symptoms are important to understand disorders such as LBP and many other musculoskeletal complaints, which demonstrate intermittent and recurrent patterns. This could cause some concern as a continuous focus on pain might be feared to affect pain perceptions and pain behaviour unfavourably, and therefore some researchers might refrain from continued monitoring. However, our results suggest that concerns related to a potential risk of negative impact are unfounded. On the other hand, as the monitoring itself may have a small positive effect on pain intensity, cohorts subjected to frequent follow-up/continued monitoring should not be regarded as untreated or representing the true natural course of a disorder. In this study no feedback was provided to patients as part of pain monitoring, and it is a topic for future research to identify useful content and formats of such feedback.

## Conclusion

We found no evidence that frequent self-reporting of pain has a negative effect on patient reported outcomes in patients with low back pain. On the contrary, we observed slightly better pain intensity outcomes after one year in patients with weekly monitoring, whereas no effects were observed on activity limitation and pain control. The role of self-monitoring as part of self-managing LBP should be investigated further.

## Data Availability

The data is available from the corresponding author on reasonable request.

## Abbreviations

ChiCo: Chiropractic Cohort
ÖMPQ: Örebro Musculoskeletal Pain Questionnaire
OPEN: Open Patient data Explorative Network
LBP: Low Back Pain
REDCap: Research Electronic Data Capture
SMS: Short Text Message
